# Shaping pathological cortical dynamics with high-frequency neurostimulation

**DOI:** 10.1186/1471-2202-16-S1-P172

**Published:** 2015-12-18

**Authors:** Jérémie Lefebvre, Micah M Murray

**Affiliations:** 1 Laboratory for Investigative Neurophysiology (The LINE), Centre Hospitalier Universitaire Vaudois, Lausanne, 1011, Switzerland; 2 EEG Brain Mapping Core, Centre for Biomedical Imaging (CIBM), 1011 Lausanne, Switzerland

## 

Disrupted neural synchrony is a hallmark pattern observed in numerous pathological states, such as dementia, depression, Parkinson's disease, epilepsy, and schizophrenia. Patients with brain lesions and conditions involving brain damage commonly display abnormal slow-wave EEG/MEG oscillatory activity that further shapes responses at rest and in response to stimuli [1]. Such pathological dysrhythmia usually takes the form of a gradual shift between fast cyclic activity in favor of slower frequency bands and strongly correlates with the degree of neurocognitive impairment [2]. Despite the diversity in the underlying pathology, dysrhythmia is a robust, consistent observation across many of these conditions, suggesting that synchrony disruption might alone be responsible of some of the symptoms. Can these defective oscillations be fixed?

In parallel and despite the surge of interest in neurostimulation techniques (e.g. DBS, TMS, tDCS) to treat brain disorders and/or manipulate brain activity, little computational insight has been gathered about the influence of electromagnetic drive on the dynamics of neural populations. Notably, the interference patterns between stimulation signals and ongoing oscillatory states are a highly debated yet still poorly understood problem [3]. How does neurostimulation interact with synchronous dynamics in the presence of pathological dysrhythmia? Can one actively tune ongoing oscillatory neural activity in order to treat neurophysiological disorders and alleviate symptoms of brain-related dysfunctions?

In this work, we analyze the influence of high-frequency stimulation on synchronous dynamics in heterogeneous networks. Using a non-linear and sparsely connected network of cortical neurons with finite conduction velocity, we examine the functional impact of local damage on network oscillatory dynamics, using both spiking and mean-field descriptions, and thus assess the influence of local neural density depletion on the genesis of pathological dysrhythmia. Our model reveals that deceleration of rhythmic activity scales with damage size, and further pairs with a concomitant decrease in spectral power. Then, building on recent findings about input-mediated tuning of synchronous oscillations and state of the art non-linear analysis tools [4], we provide insights about how weak, high-frequency neurostimulation can be used to accelerate and potentially restore oscillatory activity to healthy levels. By doing so, we propose a framework in which peculiar neurostimulation patterns, such as those accessible via TMS or tDCS used in conjunction with EEG, could bi-directionally regulate the frequency and power expressed by synchronous cortical networks. These novel findings are further shown to be distinct from the more common resonance and entrainment phenomena in which periodic stimuli enhance or even replace ongoing neural dynamics.

By addressing the implications of structural heterogeneity on synchronous dynamics and providing novel ways of compensating for them, our work outlines the key role played by axonal delays on healthy neural activity. These developments further provide key analytical and numerical insights about how pulsatile stimulation can be used to shape ongoing cyclic activity in the healthy and damaged brain.

*Financial support for this work has been provided by*: National Science and Engineering Council of Canada, Swiss National Science Foundation.
